# Simparica Trio^®^ kills *Ctenocephalides felis* on dogs and provides month-long protection against the transmission of *Dipylidium caninum*

**DOI:** 10.1186/s13071-025-07129-8

**Published:** 2025-11-27

**Authors:** Lindsay Weaver, Alta Viljoen, Riaan Maree, Lina D’Hanis, Shelby Jones, Jane Tonso, Reinier Zwiegers, Julian Liebenberg, Sean Mahabir, Keith Baker, Jessica Rodriguez, Chris Adolph, Thomas Geurden

**Affiliations:** 1 Zoetis, Veterinary Medicine Research and Development, 333 Portage St, Kalamazoo, MI 49007 USA; 2 Clinvet, Uitzich Road, Bainsvlei, Bloemfontein, 9338 South Africa; 3 Clinvet, 1479 Talmadge Hill Road South, Waverly, NY 14892 USA; 4 Zoetis, Veterinary Medicine Research and Development, Mercuriusstraat 20, 1930 Zaventem Brussels, Belgium; 5 Q-Vative, 15 Willowood, Kamoa Cresent, Wild Olive Estate, Bloemfontein, 9301 South Africa; 6 Zoetis, 10 Sylvan Way, Parsippany, NJ 07054 USA

**Keywords:** Canine, Cat flea, Cestode, Sarolaner, Simparica Trio^®^

## Abstract

**Background:**

The cestode *Dipylidium caninum* is known to infect dogs via the ingestion of an intermediate flea host, typically *Ctenocephalides felis*. Simparica Trio^®^ is an oral combination drug product for dogs effective in the treatment and prevention of fleas, including *C. felis*. Here, we report two laboratory studies evaluating the efficacy of a single administration of Simparica Trio at the minimum label dosage of 1.2 mg/kg sarolaner, 24 µg/kg moxidectin, and 5 mg/kg pyrantel (as pamoate salt) in preventing *D. caninum* infection in dogs for 1 month through killing of *C. felis*.

**Methods:**

A total of 20 dogs (*n* = 10 per group) proven to be suitable hosts for *C. felis* were used in each of the two studies. Treatment occurred on day 0, with each dog given either the placebo or Simparica Trio. On days 0 (after treatment), 7, 14, 21, and 30, dogs were infested using 200 (± 5) unfed *D. caninum*-infected *C. felis*. Live flea counts were conducted on day 33 (72 ± 2 h after day 30 infestation). All dogs were euthanized on day 58, and each dog was necropsied for the recovery of *D. caninum* scolexes from the gastrointestinal tract.

**Results:**

Placebo-treated dogs had adequate flea infestations and cestode infections in both studies. Simparica-Trio-treated dogs were free of fleas on day 33 (100% efficacy) and had significantly lower mean flea counts compared with placebo-treated dogs (*P* ≤ 0.0007). Scolex counts in Simparica-Trio-treated dogs were also significantly decreased compared with placebo-treated dogs in both studies. The efficacy of Simparica Trio against *D. caninum* based on least squares mean scolex counts was 100% (*P* < 0.0001) in study 1 and 92.1% (*P* = 0.0033) in study 2.

**Conclusions:**

The efficacy provided by Simparica Trio against *C. felis* at the minimum dosage of 1.2 mg/kg sarolaner, 24 µg/kg moxidectin, and 5 mg/kg pyrantel (as pamoate salt) prevented *D. caninum* infection in dogs for 1 month.

**Graphical Abstract:**

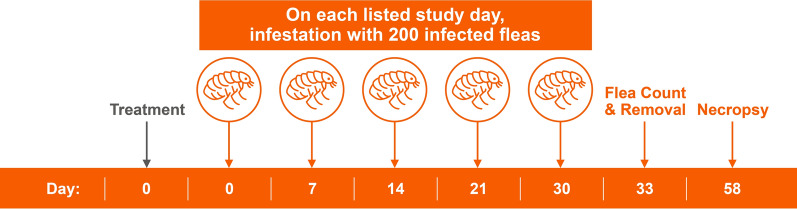

## Background

The cat flea, *Ctenocephalides felis*, is one of the most important flea species globally in terms of human and veterinary health and the species typically most prevalent on domestic dogs [1, 2]. Direct effects of canine *C. felis* infestations include the immediate irritation experienced in response to flea bites and the possible development of flea allergy dermatitis (FAD) in susceptible dogs exposed to recurring flea feeding [3–5]. Another less evident but significant threat associated with *C. felis* infestation is that of disease transmission, as the flea species is known to act as a vector for multiple pathogens [6]. For dogs, infestation with *C. felis* brings with it the risk of infection with *Dipylidium caninum*, as this flea species is the most prevalent intermediate host of this canine cestode and the primary source of dipylidiasis in domestic dogs [3, 4, 7–14]. Dogs become infected with *D. caninum* when they ingest fleas carrying the infective cysticercoid life stage, with adult tapeworms residing in the intestine and releasing egg packets into the environment via the dog’s feces [14–18].

As *D. caninum* is zoonotic, infected *C. felis* fleas on dogs and in the home expose people, especially children, at risk of infection, with prevalence in humans generally considered low but also probably under-detected [17, 19–22]. Both human and canine dipylidiasis can be effectively treated, but to truly interrupt the *D. caninum* lifecycle and prevent reinfection it is necessary to also kill the intermediate flea host. Simparica Trio^®^ is a safe oral combination drug product formulated to deliver a minimum dose of 1.2 mg/kg sarolaner, 24 µg/kg moxidectin, and 5 mg/kg pyrantel (as pamoate salt) and proven to be effective in the treatment and prevention of canine *C. felis* infestations [23, 24]. The studies reported here were designed to investigate whether the efficacy against *C. felis* infestation on dogs would also prevent canine *D. caninum* infection.

## Methods

The two studies were placebo-controlled laboratory efficacy studies. Each study was conducted by Clinvet either in New York, USA (study 1) or Bloemfontein, South Africa (study 2). A licensed veterinarian supervised the care of all dogs for the duration of each study, and the studies complied with all applicable local, state laws, and national regulations related to the humane care and use of animals. All study protocols were approved by the Study Site Institutional Animal Care and Use Committee, and all study procedures were in accordance with the relevant World Association for the Advancement of Veterinary Parasitology guidelines [25–27].

**Figure 1:**
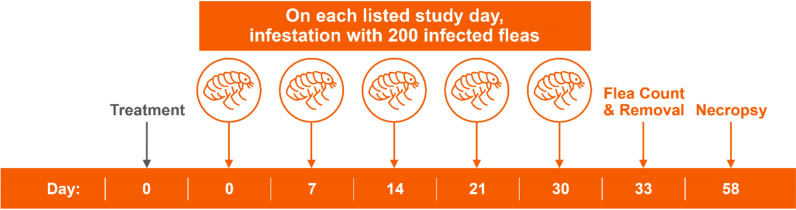
Study design used in study 1 and 2

### Design

A fecal sample collected from each dog (*n* = 24) on day −9 was tested for *D. caninum* using a polymerase chain reaction (PCR) assay [16] and all dogs were comb-counted on day −8 to ensure they were free of fleas. Host suitability of the 24 dogs was determined in each study by infesting each dog on day −8 with either 100 (± 5) (study 1) or 200 (± 5) (study 2) viable, unfed adult *C. felis* (not infected with *D. caninum*). On day −7 (24 h post-infestation), all dogs were examined and comb-counted, and any identified fleas were removed. The 20 dogs with the highest flea counts were selected for each study and administered 5 mg/kg of praziquantel prior to day −7 to treat possible undetected *D. caninum* infection prior to randomization. Dogs were allocated to treatments and cages according to a randomized complete design with blocking based on pre-infestation flea counts. Blocks consisted of two dogs, with one animal randomized to each treatment within the block. Dogs enrolled in both studies were moved to their allocated cages on day −1. All dogs were treated on day 0 with either placebo (Pet-Tabs^®^ tablet) or Simparica Trio. All dogs were infested with 200 (± 5) viable, unfed adult *D. caninum*-infected *C. felis* fleas on days 0 (post-treatment), 7, 14, 21, and 30 (Fig. 1). Dogs were allowed to engage in normal grooming behavior throughout the course of the study to mimic the natural infection method of *D. caninum*. Live flea counts were conducted on day 33, with removal of the fleas. All dogs were euthanized on day 58 and necropsies were performed to recover, identify and count *D. caninum* scolexes from the gastrointestinal tracts of each dog.

Masking was accomplished by separation of functions of study personnel. All persons making observations; performing PCR testing; conducting scolex counts, post-treatment flea infestations, and post-treatment flea counts; or performing general care for the dogs were masked to experimental treatments.

### Animals

A veterinarian experienced in general canine medicine confirmed all enrolled dogs were clinically normal. Exclusion criteria included being fractious, suffering from disease or injury, or otherwise unsuitable for inclusion according to an experienced veterinarian.

Purpose-bred dogs from the Clinvet colonies were enrolled in each study with study 1 consisting of beagles and study 2 of mixed breed mongrels. All enrolled dogs underwent a physical examination on day −8 that included, but was not limited to, rectal temperature, thoracic auscultation, skin and hair coat assessment, and an assessment of the general physical condition of each dog. All dogs were housed in individual cages such that no physical contact between dogs was possible, and they were fed commercially available dry food once per day, with fresh water provided ad libitum. General health observations were performed twice per day from day −14 through day 58 with the following exceptions: general health observations were made once on day 1 (separate to the clinical observations made on this day), once on day 58 (prior to euthanasia), and were not made on the day physical examination was performed (day −8) or on day 0 when multiple clinical observations were performed.

### Flea infestations and assessment

Study 1 used viable, unfed adult *C. felis* sourced from a colony at Ecto Services, Inc (Henderson, NC, USA). This colony, originally obtained in April 2020, was enriched with fleas from Henderson, NC in July 2022. Study 2 used viable, unfed adult *C. felis* fleas from a colony sourced from PLRS laboratories (Corapeake, NC, USA) in 2010 and enriched with fleas from Sierra Research Laboratories (Modesto, CA, USA) in 2017. All fleas were infected with *D. caninum*, originally collected from cats and dogs in the field in Oklahoma, USA in 2015 (study 1) or from cats in Thessaloniki, Greece in 2019 (study 2), as follows: freshly collected flea eggs were added to *D. caninum* proglottids for a period of 3 days. After the flea eggs hatched, the resulting larvae are exposed to the proglottids and were thereby forced to feed on them. These flea larvae were then transferred to a standard flea-breeding medium, where they were allowed to complete their developmental cycle. The fleas were confirmed to be infected with *D. caninum* at an infection rate ranging from 10.0% to 30% in study 1 and from 10.0% to 36.67% in study 2. Each dog was infested with 100 (± 5) (study 1) or 200 (± 5) (study 2) viable, unfed adult *D. caninum*-free *C. felis* fleas prior to study start to determine host suitability. Each dog was infested with 200 (± 5) viable, unfed adult *D. caninum*-infected *C. felis* fleas on days 0, 7, 14, 21, and 30. A vial containing 200 (± 5) fleas was swirled to form a flea pellet, which was then deposited onto the dog’s hair coat. The vial was held in position for ~10 s to ensure the flea pellet was not dislodged and to facilitate dispersal of the fleas into the hair coat. The container was gently removed after all the fleas had disappeared into the hair coat.

Live flea counts with removal of the fleas were conducted on day 33. Each dog was removed from its cage for counting. Fine-toothed flea combs were used to comb the entire body of the dog, starting with the anterior end and working toward the posterior end and ventral areas. Any collected fur was removed from the comb and separated, and fleas within the fur were counted. Dogs were combed for at least 10 min, and if fleas were encountered in the last 5 min, combing continued in 5-min increments until no fleas were encountered. Personnel conducting counts were masked to treatment assignments and changed gloves between each dog.

All dogs were euthanized on day 58 following a fasting period of 15–18 h. The gastrointestinal tracts were removed and ligated to separate the stomach, duodenum, small intestines, large intestines, and the end of the rectum. Each section was cut open, and the mucosa was observed macroscopically for the presence of worms, which were collected and preserved. The mucosa was washed with water and scraped, with the washings and scrapings collected separately washed through 0.150 mm sieves. The collected material was examined using a stereomicroscope, and the number of scolices were counted and recorded.

### Treatment and observations

Clinical observations were performed on all dogs prior to treatment on day 0. All dogs were then dosed orally on day 0 with either one half or a full Pet-Tabs tablet (T01, placebo) or Simparica Trio (T02; 1.2 mg/kg sarolaner, 24 µg/kg moxidectin, and 5 mg/kg pyrantel (as pamoate salt)). To assist swallowing, each dog was administered approximately 5 mL of water by mouth via syringe. Dogs were periodically observed for several minutes after dosing for evidence that the dose was swallowed and for potential adverse events (e.g., choking, drooling, gagging, or vomiting). Approximately 2 h after dosing, all dogs were observed and pens inspected for evidence of emesis or expelled test product. After treatment on day 0, clinical observations were performed at 1 h (± 15 min), 3 h (± 30 min), 6 h (± 1 h) and 24 h (± 1 h) after dosing.

### Statistical analysis

Challenge was considered adequate if 60% of the placebo-treated control dogs maintained ≥ 50 fleas following the final flea infestation on day 30 and 60% of the placebo-treated control dogs had ≥ 2 adult *D. caninum* at the time of necropsy [25]. The experimental unit was the individual dog. No data collected from incompletely dosed animals were included in the analyses. Scolex counts were the primary efficacy variable. Both scolex count and flea counts were transformed with a natural logarithm transformation [loge(x + 1)] prior to analysis with a general linear mixed model containing the fixed effect of treatment and the random effects of room and block within room. Treatment least squares means, standard errors, 95% confidence limits, and minimums and maximums were calculated. Treatment arithmetic means were calculated (back-transformed arithmetic means for log-transformed flea counts, [loge(x + 1)]). For scolex counts, the least squares means, standard errors, and confidence limits were back-transformed for presentation. For both scolex and flea counts, the treatments were compared, and efficacy was calculated as follows: 100 × (control mean–Simparica Trio mean)/control mean.

## Results

### Dosing and observations

Study 1 utilized 10 males and 10 females aged 20–50 months at study start and weighing 7.3–14.5 kg on day −1. Study 2 utilized 4 males and 16 females aged 21–95 months of age at study start and weighing 12.7–28.6 kg on day −1. Dogs were intact or neutered, none were pregnant or lactating, and all were uniquely identified. All dogs had been regularly dewormed and previously vaccinated as required, but no dog was given medication from day −14 through study end (unless concomitant treatment was required to maintain adequate care). All dogs in study 1 received a complete dose of either placebo or Simparica Trio, with no abnormal clinical observations reported for dogs on day 0. In study 2, 19 of the 20 dogs received a complete dose of allocated treatment, with one dog in the placebo group underdosed due to partial regurgitation within 1 h of administration. This dog was excluded from data analyses. One dog in the placebo group had gastritis from day 7 until day 10, which was treated with Pro-Kolin (probiotic) and ulsanic acid (sucralfate 1 g/5 mL) (both 5 mL orally twice daily for 5 days) and resolved. One dog in the Simparica Trio group had mild diarrhea on day 0 (not considered treatment related) that required no concomitant treatments and resolved the same day. A second dog in the Simparica Trio group was noted to be lethargic prior to dosing, was examined thoroughly and confirmed fit to continue the study. No concomitant treatments were required, and the clinical observation resolved the same day. No dogs in either study were reported to have post-treatment clinical observations of ataxia, panting, salivation, sedation, seizures, or shivering.

### Flea counts

Placebo-treated control dogs maintained adequate infestations throughout the study, with 70% of placebo-treated dogs in both studies having ≥ 50 fleas on day 33. On that day, flea counts in the placebo group ranged from 5 to 127 in study 1 and from 20 to 159 in study 2. All Simparica-Trio-treated dogs were free of fleas on day 33, and there was a statistically significant decrease in mean flea counts from Simparica-Trio-treated dogs compared with placebo-treated dogs (*P* ≤ 0.0007) in both studies (Table 1). Efficacy based on least squares mean flea counts was 100% in both studies.

**Table 1:** Mean live *Ctenocephalides felis* counts and ranges for dogs treated with a single dose of Simparica Trio^®^ and percent efficacy relative to placebo

Study	Treatment^1^	Dogs infested^2^ (n)	Arithmetic mean	Least squares mean	Range	Standard error	95% confidence limits	Efficacy^3^ (%)	*P*-value^4^
1	Placebo	10	67.0	67.0	5–127	9.49	45.4–88.5	–	< 0.0007
	Simparica Trio	0	0.0	0.0	0–0	9.49	−21.5–21.5	100	
2	Placebo	9	96.4	94.7	20–159	18.87	51.2–138.3	–	< 0.0001
	Simparica Trio	0	0.0	0.0	0–0	18.64	−43.0–43.0	100	

^1^*n* = 10 for all groups except *n* = 9 for placebo in study 2 due to one animal receiving an incomplete dose

^2^A total of 7 dogs in the placebo-treated control group had ≥ 50 fleas in each study

^3^Efficacy based on least squares means used as arithmetic means relative to placebo on day 33. Least squares means used to adjust for the unequal replication for treatments in study 2

^4^Significant difference (*P* ≤ 0.05) between treatment groups

### Scolex counts

Day 58 scolex counts showed 90% (study 1) and 88.9% (study 2) of placebo-treated control dogs had ≥ 2 *D. caninum* scolexes, indicating adequate infections for efficacy evaluation. Scolex counts in *D. caninum*-infected placebo dogs ranged from 1 to 100 in study 1 and 1–54 in study 2 (Table 2). In contrast, no Simparica-Trio-treated dogs in study 1 were infected with *D. caninum*, and only three were infected with *D. caninum* in study 2 (with a maximum scolex count of 3). Of note, *D. caninum* proglottids with egg packets (but no scolexes) were recovered from one dog in each treatment group in study 2; each of these dogs were counted as *D. caninum* infected. In both studies, scolex counts in Simparica-Trio-treated dogs were significantly decreased compared with placebo-treated dogs (*P* ≤ 0.0033; Table 2), and the efficacy of Simparica Trio based on least squares mean scolex counts in both studies was ≥ 92.1%.

**Table 2:** Mean *Dipylidium caninum* counts and ranges for dogs administered a single dose of placebo or Simparica Trio^®^ and percent efficacy relative to placebo

Study	Treatment^1^	Dogs infected^2,3^ (n)	Arithmetic mean	Range	Back-transformed			Efficacy^4^ (%)	*P*-value^5^
					Geometric mean	Standard error	95% confidence limits		
1	Placebo	10	25.8	1–100	15.9	4.05	8.8–28.1	–	< 0.0001
	Simparica Trio	0	0.0	0–0	0.0	0.24	0.0–0.7	100	
2	Placebo	9	9.7	1–54	5.2	1.9	2.11–11.6	–	0.0033
	Simparica Trio	3	0.7	0–3	0.4	0.3	− 0.1–1.2	92.1	

^1^*n* = 10 for all groups except *n* = 9 for placebo in study 2 due to one animal receiving an incomplete dose

^2^Study 1, nine (90%) dogs in the placebo group had ≥ 2 scolexes; study 2, eight (88.9%) dogs in the placebo group had ≥ 2 scolexes, and two dogs in the Simparica Trio group had ≥ 2 scolexes

^3^In study 2, *D. caninum* proglottids containing egg packets (but no scolexes) were recovered from one dog in the placebo group and one dog in the Simparica Trio group

^4^Efficacy based on geometric mean (back-transformed least squares mean) relative to placebo

^5^Significant difference (*P* ≤ 0.05) between treatment groups

## Discussion

The efficacy of Simparica Trio against *C. felis* recorded in the two studies described here aligns with previously reported data [23, 24], with one administration at minimum label dose (1.2 mg/kg sarolaner, 24 µg/kg moxidectin, and 5 mg/kg pyrantel (as pamoate salt)) providing 100% protection against flea infestations for 1 month. In contrast, *C. felis* were recovered from all placebo-treated dogs on day 33, with ≥ 70% of control dogs in each study having at least 50 fleas. The successful management of *C. felis* is essential to the health and well-being of domestic dogs,. As a host-generalist and a carrier for various pathogens, *C. felis* contributes to parasite spillover among wild animals, domestic animals, and humans [3, 12, 28–32], such that effective control of the flea species can prevent or reduce the impact of other diseases of veterinary and medical importance [33]. These two studies were based on the rationale that the killing of *C. felis* using Simparica Trio would translate to protection against the transmission of *D. caninum* to dogs [33, 34], and the data from these two studies confirm this reasoning to be valid. Scolex counts from placebo-treated dogs confirmed that adequate *D. caninum* infections were achieved, with all placebo-treated dogs positive for *D. caninum*, and ≥ 88.9% in each study having at least two scolexes (scolex counts ranged from 1 to 100). However, necropsies of Simparica-Trio-treated dogs revealed either none (study 1) or only three (study 2) were positive for *D. caninum*, with parasite counts ranging from 0 to 3. These data confirm that through its efficacy against *C. felis*, a single oral dose of Simparica Trio at minimum label dose also afforded dogs with 30 days of protection against the transmission of *D. caninum*.

## Conclusions

The efficacy afforded by a single dose of Simparica Trio at the minimum dosage of 1.2 mg/kg sarolaner, 24 µg/kg moxidectin, and 5 mg/kg pyrantel (as pamoate salt) against *C. felis* prevented the transmission of *D. caninum* to dogs for 1 month.

## Data Availability

The data supporting the conclusions of this article are reported within the article.

## Abbreviation

USA: United States of America
